# Peritonectomy for disseminated peritoneal implantation after dermoid cyst spillage causing recurrent peritonitis and xanthogranulomatous reaction: a case report

**DOI:** 10.1093/jscr/rjaf739

**Published:** 2025-09-13

**Authors:** Melanie Mercado, Ashish Vaska, Joseph Chen, Salman Marvi, Ruwanthi Wijayawardana, Nima Ahmadi, David Morris

**Affiliations:** Department of Surgery, Hepatobiliary and Surgical Oncology Unit, St George Hospital, Gray St, Kogarah NSW 2217, Australia; Department of Surgery, Hepatobiliary and Surgical Oncology Unit, St George Hospital, Gray St, Kogarah NSW 2217, Australia; Department of Anatomical Pathology, St George Hospital, Gray St, Kogarah NSW 2217, Australia; Department of Anatomical Pathology, St George Hospital, Gray St, Kogarah NSW 2217, Australia; Department of Surgery, Hepatobiliary and Surgical Oncology Unit, St George Hospital, Gray St, Kogarah NSW 2217, Australia; Department of Surgery, Hepatobiliary and Surgical Oncology Unit, St George Hospital, Gray St, Kogarah NSW 2217, Australia; Department of Surgery, Hepatobiliary and Surgical Oncology Unit, St George Hospital, Gray St, Kogarah NSW 2217, Australia; St George and Sutherland Clinical School, University of New South Wales, Anzac Pde, Kensington NSW 2033, Australia

**Keywords:** chronic peritonitis, dermoid cyst, mature cystic teratoma, peritonectomy

## Abstract

Dermoid ovarian cysts, or mature cystic teratomas, are common benign ovarian masses. Surgical intervention for symptomatic cysts includes minimally invasive or open cystectomy. The consequences of intra-abdominal spillage during cystectomy can be significant and include acute chemical peritonitis or chronic granulomatous inflammatory reactions. This is the first reported case of peritonectomy for disseminated dermoid disease. This case report discusses a previously healthy 20-year-old patient who developed both these complications following elective laparoscopic ovarian cystectomy for dermoid ovarian cysts. She required multiple admissions and a multi-disciplinary approach to develop a treatment plan, culminating in peritonectomy. This case necessitated significant multi-disciplinary discussion to address complex treatment decisions regarding anti-microbial and surgical management. This is the first reported case of peritonectomy for source control of chronic sepsis and granulomatous peritonitis following disseminated dermoid disease, highlighting the importance of timely intervention and thorough peritoneal wash-out to mitigate late inflammatory complications.

## Introduction

Dermoid ovarian cysts, or mature cystic teratomas, are among the most prevalent benign ovarian neoplasms, representing ~10%–25% of all ovarian tumours [1]. Although dermoid cysts can manifest at any age, they are most commonly diagnosed in women between the ages of 20 and 40 years. These cysts develop from germ cells and are typically composed of a variety of tissues, including skin, hair, teeth, and sometimes fat or muscle. Known risk factors for dermoid cyst formation include a familial history of ovarian tumours, prior pelvic surgery, and endocrine disorders such as polycystic ovary syndrome that may affect ovarian function. While often remaining asymptomatic, they can lead to clinical complications, such as torsion, rupture, or infection [1].

Management of mature cystic teratomas depends on the presence and severity of symptoms, the size of the cyst and the risk of complications such as torsion or rupture. For symptomatic or complicated lesions, surgical intervention is generally indicated. In cases where the cyst is large, complicated by torsion, or suspicion for malignancy, an oophorectomy may also be performed. In all instances, surgical intervention aims to remove the cyst while preserving ovarian function, especially in younger patients desiring fertility preservation. Laparoscopic cystectomy has become the preferred approach, offering the advantages of smaller incisions, quicker recovery, and reduced postoperative complications [2]. However, a laparoscopic approach has an 11%–16% rate of conversion to laparotomy, higher rate of spillage compared with laparotomy [3] and higher risk of recurrence [4].

## Case history

A 20-year-old female underwent an elective laparoscopic bilateral ovarian cystectomy for large ovarian cysts (one left sided, 20 cm and two right sided, 5 and 10 cm). She had no other medical or surgical history. Intraoperatively, there was rupture and spillage of a right sided cyst. The abdomen was washed out with 5–7 L of fluid prior to closure and the patient was discharged the following day after observation. Histopathology confirmed benign dermoid cysts.

After her index operation, the patient had five admissions for abdominal pain and intra-abdominal sepsis, including an admission to the Intensive Care Unit for septic shock, diagnostic laparoscopy, and washout and drainage of an abdominal wall abscess. The infectious diseases (ID) team was extensively involved in both the inpatient and outpatient settings.

**Figure 1:**
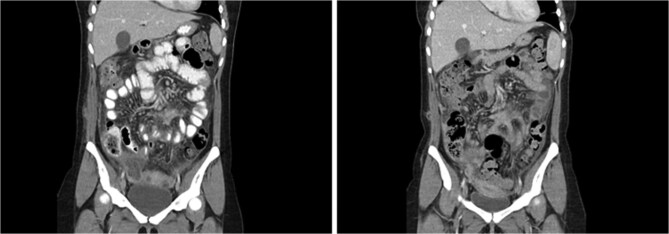
Coronal CT abdomen from admission 3 (left) and admission 4 (right). Heterogenous collection in right pouch of Douglas, progression of mesenteric/omental fat stranding, thickened loops of small bowel.

Due to the unexpected severe and prolonged inflammatory reaction to dermoid cyst spillage (Fig. 1), the immunology team were consulted during admission 4. The option of empiric immunosuppression with prednisolone was discussed between the ID, immunology and treating teams but given the absence of an underlying autoimmune or autoinflammatory condition, the risk of sepsis and the lack of evidence supporting immunosuppression, this was not pursued. However, the patient remained symptomatic with ongoing abdominal pain and persistent fevers.

At this stage, the option of a peritonectomy to obtain definitive source control was raised with the patient. After further consultation with medical oncology and ID, the patient proceeded with a peritonectomy. Intra-operative findings included extensive dermoid seeding and hairs from the cyst throughout the peritoneum with significant inflammatory change and pockets of infected fluid. A large dermoid mass involving omentum, terminal ileum, caecum, and sigmoid colon was found in the pelvis and both fallopian tubes were grossly abnormal, dilated and adherent to the pelvic mass. There was thick disease overlying the liver surface and lower abdominal wall. An intra-operative consult from the original gynaecologist recommended salpingectomy due to the risk of ectopic pregnancy and preservation of the ovaries. The operation involved an extensive adhesiolysis, parietal and pelvic peritonectomy, bilateral diaphragm stripping, omentectomy, ileocolic resection, anterior resection, resection of ileum segment involved in dermoid mass, and bilateral salpingectomy.

**Figure 2:**
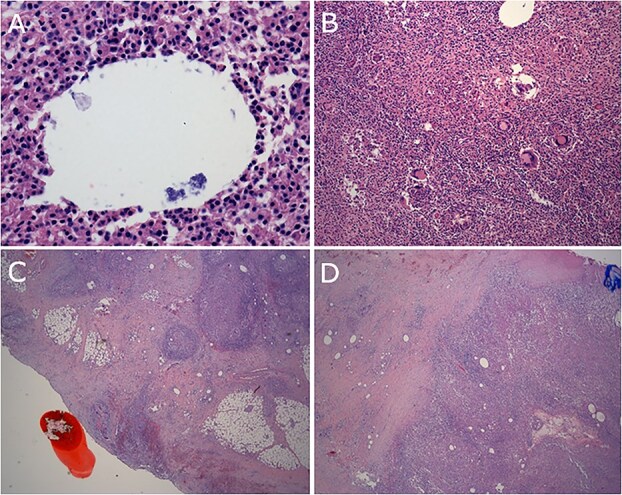
(A) Bacterial organisms within vacuole. (B) Granulomas and giant cells. (C) Reaction involving serosal surface of colon. (D) Reaction involving diaphragm.

Final histopathology reported extensive xanthomatous and suppurative granulomatosis reaction to dermoid cyst involving all resected organs and large and small bowel microperforations (Fig. 2). Cultures from intra-operative swabs grew Candida and *Enterococcus faecium*. After discharge the patient followed up with fertility specialists who performed egg collection.

She continued to have intermittent abdominal pain requiring regular opioid analgesia. She trialled oral prednisolone for 6 months without improvement to pain. Progress computed tomography (CT) imaging had shown progression of residual disease in the pelvis and subcapsular hepatic disease. Steroids were subsequently ceased. One year following her first peritonectomy, she proceeded to a repeat peritonectomy which found dermoid deposits on small bowel mesentery, grossly abnormal ovaries with residual dermoid material including hair in the pouch of Douglas, and liver surface disease. The operation involved bilateral oophorectomy, removal of peritoneal nodules and ablation of liver surface disease. Histopathology again showed granulomatous inflammation and hair shaft fragments. Her post-operative recovery was uncomplicated and she was discharged home (Day 15).

## Discussion

Ruptured dermoid ovarian cysts and the subsequent spillage of their contents into the abdominal cavity can pose significant clinical challenges and potential complications as demonstrated in this case. The most common cause of rupture is idiopathic while other causes include torsion, trauma, pregnancy or iatrogenic [2]. The introduction of foreign material into the peritoneal space can provoke an acute or chronic inflammatory response, resulting in chemical peritonitis or granulomatous reaction. Moreover, contamination with dermoid contents can significantly heighten the risk of postoperative infections, leading to prolonged hospitalization and impaired wound healing. Long-term complications include chronic pain, bowel obstruction, abscess formation, and enterocutaneous fistula formation [5].

The treatment of spillage involves control of the contaminated area with thorough lavage and suction of the peritoneal cavity to dilute and remove any residual material, reducing the risk of peritoneal reaction [6]. It is critical to evaluate the extent of spillage and ensure that no viable tissue, particularly hair or sebaceous material, is left within the peritoneal space. Post-operative management includes vigilant monitoring for signs of infection or peritoneal irritation, along with the administration of broad-spectrum antibiotics to prevent secondary infection, where indicated. While there are reports of patients with residual chronic granulomatous peritonitis being treated with immunosuppression, the evidence supporting its role is lacking and it carries a high risk of sepsis-related complications [2].

In this case, following iatrogenic spillage of cyst contents, the patient developed a chemical peritonitis, and despite a laparoscopic washout, developed an established granulomatous peritonitis with chronic sepsis. This case necessitated significant multi-disciplinary collaboration to address complex treatment decisions regarding anti-microbial and surgical management. This is the first reported case of peritonectomy for source control of chronic sepsis and granulomatous peritonitis following disseminated dermoid disease, highlighting the importance of timely intervention and thorough peritoneal wash-out to mitigate late inflammatory complications.
